# Understanding factors related to application of traditional Chinese medicine tuina for congenital muscular torticollis in children: a qualitative study based on traditional Chinese medicine tuina practitioners

**DOI:** 10.1186/s12906-026-05246-0

**Published:** 2026-01-10

**Authors:** Xuan Zhang, Shu-Cheng Chen, Wing-Fai Yeung, Fei-Fei Ding, Ya-Zheng Pang, Yan Sun, Jing Wu, Juan Yu

**Affiliations:** 1 Affiliated Hospital of Shandong University of Traditional Chinese Medicine, 16369 Jing Shi Road, Jinan, Shandong China; 2 The Hong Kong Polytechnic University, Hong Kong, China

**Keywords:** Tuina, Congenital muscular torticollis, Qualitative research, Tuina practitioners

## Abstract

**Background:**

Congenital muscular torticollis (CMT) is a common pediatric congenital musculoskeletal abnormality that may impact children’s development even quality of life if not managed appropriately. Traditional Chinese Medicine (TCM) tuina is a manual therapy derived from the theoretical foundations of TCM, has become an important non-invasive intervention in pediatric clinical practice, particularly for the management of CMT. However, the factors influencing its application remain insufficiently explored. Understanding these factors from the perspective of TCM tuina practitioners is crucial for optimizing treatment strategies and promoting broader integration of TCM tuina in pediatric care. This study aims to explore key factors related to the clinical application of TCM tuina for children with CMT, in order to inform clinical practice and support the more effective implementation of tuina therapy in pediatric care, based on qualitative insights from experienced TCM tuina practitioners.

**Method:**

A qualitative research design was employed, with data collected from a purposive sample of 15 practitioners, each with over three years of experience in TCM tuina at three different public hospitals. Individual interviews were conducted using a semi-structured interview guide. The data were processed using template analysis, facilitated by MS Word software. The Consolidated Criteria for Reporting Qualitative Research (COREQ) checklist guided this study’s reporting.

**Results:**

Two main themes included: (1) existed problems, (2) optimized approaches. The theme of existed problems included the subthemes of: (a) Variability in clinical practice, (b) Barriers to parental involvement, (c) Importance of strong parental participation in TCM tuina. The theme of optimized approaches included the subthemes of: (a) Promotion of public awareness, (b) Improvement of treatment protocols, (c) Enhancement of practitioner–parent communication.

**Conclusion:**

This study highlights key factors influencing the clinical application of TCM tuina for children with CMT, as identified by experienced practitioners. Challenges such as inconsistent treatment practices, limited parental awareness, and inadequate family involvement were commonly reported. Practitioners emphasized the importance of early intervention, individualized tuina techniques, standardized protocols, and active parent-practitioner collaboration. These findings provide valuable insights for optimizing treatment strategies and promoting the effective integration of TCM tuina into pediatric care for CMT.

**Supplementary Information:**

The online version contains supplementary material available at 10.1186/s12906-026-05246-0.

## Background

Congenital muscular torticollis (CMT) is one of the most common congenital musculoskeletal abnormalities in newborns, second only to congenital hip dysplasia and clubfoot. The global incidence of CMT varies from 1% to 3.92% [1–3], with some studies indicating it occurs in at least one in every 250 newborns [4]. The condition is commonly associated with fibrosis of the sternocleidomastoid muscle (SCM). If left untreated, CMT can progressively lead to permanent torticollis, resulting in complications such as craniofacial asymmetry, impaired eye movement, visual deficits, neck discomfort, spinal curvature, and other related symptoms [5–8]. These complications can significantly impact a child’s growth and development, posing serious challenges to their overall well-being and quality of life [9].

The treatment of CMT includes both invasive interventions (e.g., botulinum toxin injections or surgery) and conservative therapies [10, 11]. Invasive interventions are typically considered when conservative treatments fail to show progress after six months or if the child begins treatment after the age of one with significant limitations and/or an SCM mass. Postoperative management of CMT generally requires at least four weeks, or longer, for scar care, muscle strengthening, and range of motion recovery [12, 13]. Conservative treatment includes manual therapy, external treatment of herbal medicine, muscle strengthening exercises, microcurrent therapy, kinesiological taping, tubular orthosis and so on [10, 11, 14, 15]. Among these, manual therapy represents a low-risk intervention with potential therapeutic benefits for CMT and may be a reasonable option for parental consideration [16–19].

Traditional Chinese medicine (TCM) tuina, a specialized form of conservative therapy rooted in TCM theory, involves the skilled application of manipulations such as pushing, kneading, and holding on specific acupoints or body areas [20–22]. Unlike general TCM approaches, which may include herbal medicine, acupuncture, or moxibustion, tuina therapy focuses specifically on physical manipulation to achieve therapeutic effects. Studies have shown that through TCM tuina manipulation, it is possible to induce telangiectasia, improve blood circulation, and enhance the nutritional status of muscle tissue at the site of the lesion. These effects collectively contribute to the promotion of muscle tissue recovery [21, 23–26].

TCM tuina is a commonly used non-invasive manual therapy in China, widely applied in pediatric care [27], especially in the management of CMT [22]. In over 50% of maternity and child health hospitals in China, which are equipped with TCM departments, the role of tuina in daily clinical practice has become increasingly prominent [28]. However, the clinical application of TCM tuina is influenced by multiple contextual factors, many of which are directly observed by tuina practitioners in daily practice. To date, little is known about how TCM tuina is applied in clinical settings and how practitioners address these challenges. In particular, the experiences and perspectives of frontline TCM tuina practitioners have been largely overlooked. This qualitative study aims to explore the practical experiences and perspectives of TCM tuina practitioners in managing pediatric CMT, in order to better understand real-world clinical applications and challenges, and inform future implementation strategies.

## Methods

### Study design

This research employed a qualitative research approach, using semi-structured, face-to-face individual interviews. The research adhered to the Standards for Reporting Qualitative Research [29]. The interviews were recorded with the participants’ consent. We followed the Consolidated Criteria for Reporting Qualitative Research (COREQ) checklist [30] to ensure comprehensive reporting of qualitative research.

### Participants and sampling

This study aims to explore factors influencing the application of TCM tuina for CMT in children, based on the perspectives of TCM tuina practitioners. A purposive sampling strategy was employed to recruit 15 TCM tuina practitioners from Grade A tertiary hospitals across northern and southern mainland China. In China, Grade A tertiary hospitals are top-tier medical institutions known for excellence in clinical practice, research, and education, making practitioners well-qualified for this study. A sample of 15 participants was deemed sufficient, as it fully satisfied the widely accepted criterion of 12 for reaching data saturation in qualitative research, proposed by Lincoln and Guba [31] and supported by Vasileiou et al. [32]. Individuals or the offices where they work were called to invite selected professionals to participate. The inclusion criteria were: (a) working as TCM tuina practitioners with a minimum of three years’ clinical experience, (b) having treated CMT in at least 20 cases, including both mass and non-mass type, with demonstrated initial therapeutic recovery (c) proficient in Mandarin for effective communication, and (d) willing to sign the informed consent form. All recruited practitioners confirmed having successfully treated more than 8 cases of both mass type and non-mass type CMT, ensuring adequate clinical experience across CMT subtypes.

### Data collection

A semi-structured interview guide was drafted and revised based on relevant literature and discussions with two experts in the field of TCM tuina and one qualitative researcher (SCC). We focused on clinical trials and qualitative literature related to TCM tuina, particularly in pediatric conditions. This informed the development of our interview guide and enabled us to formulate questions that deeply explored the perspectives of tuina practitioners managing CMT. In addition, we reviewed literature concerning the clinical practice of TCM tuina, treatment adherence, and decision-making processes, thereby deepening our understanding of the factors influencing practitioners’ experiences in applying TCM tuina in the treatment of CMT. These insights guided the refinement of our interview guide, allowing for an in-depth exploration of factors contributing to variations in treatment outcomes among tuina practitioners treating CMT. This strategy ensures our study captures detailed, context-rich data and contributed to addressing identified gaps noted in prior research. A pilot version was subsequently developed and revised by two TCM practitioners (XZ and YS) and a qualitative research expert (WFY). It was then pre-tested through two pilot interviews to evaluate the clarity and appropriateness of the questions. The final version consisted of 10 questions and is presented in Table 1. The interview questions were designed to elicit a comprehensive understanding of TCM tuina therapy as applied in clinical practice, situated within the broader context of CMT diagnosis, treatment, and caregiver involvement. For instance, Q1–Q3 explored how practitioners identify, classify, and diagnose CMT—critical foundations for selecting TCM tuina interventions. Q4–Q6 examined treatment environments, clinical goals, and practitioners’ perceptions of TCM tuina efficacy. Q7–Q9 addressed practical challenges, including caregiver adherence, communication, and concurrent or alternative therapies. Q10 considered the broader application of tuina during or after CMT treatment. Although not all questions explicitly mentioned TCM tuina, they were intentionally designed to capture practitioners’ perspectives into its clinical application. This approach aligns with the exploratory aims of qualitative research in understanding real-world clinical reasoning and practices.

**Table 1:** Questions for the semi-structured interview

1. Would you please introduce the types of CMT that you have seen in your clinic?
2. Do you think that all types of CMT are suitable for pediatric massage therapy? Are there any situations that don’t fit?
3. In your clinical diagnosis and treatment of muscular torticollis, what signs or examination results do you usually use to diagnose muscular torticollis?
4. What kind of medical environment do you think is more suitable for the treatment of CMT?
5. What are the TCM tuina manipulations you usually use to treat CMT? How long does each manipulations apply?
6. What are the criteria you use to determine the cure of CMT in children?
7. How many of these patients do you receive adhere to treatment to heal? Among them, what do you think is the probable cause for patients who give up or interrupt treatment?
8. Do you think it is necessary for parents to cooperate during the treatment of children? What are the specific aspects?
9. Based on your experience and expertise, what other treatment options are available to treat pediatric muscular oblique neck? What conditions do you recommend for treatment?
10. Will CMT children seek treatment for other diseases or conditioning physique during or after treatment of muscular oblique neck?

*CMT* Congenital muscular torticollis, *TCM* Traditional Chinese medicine

Face-to-face, semi-structured interviews were conducted by XZ (the moderator of the individual interviews) and FFD (the assistant moderator) in a private room at the hospital. Participants were given the flexibility to select the timing that best accommodated their schedules. For training purposes, SCC listened to one interview conducted by XZ and FFD, followed by discussion to optimize interview technique. Before the official interviews began, XZ briefly introduced themselves to establish a connection with the participants and provided a comprehensive explanation of the research’s purpose, significance, and methodology. Throughout the interviews, FFD was responsible for taking notes and managing the voice recorders. Participants were offered a $50 cash to compensate them for their time. All interviews were recorded digitally and transcribed verbatim by either first author or by a transcriber, who had previous experience of transcribing qualitative interviews. Finally, transcripts were returned to interviewees for comments. Thematic saturation was assessed continuously during the data collection process. After every 2–3 interviews, XZ and SCC reviewed the transcripts and conducted preliminary manual coding using Microsoft Word software [33, 34]. Codes were highlighted and categorized to track the emergence of new concepts. While new themes continued to develop during the early and middle phases, no additional themes were identified after the 13th interview. The last two interviews served to confirm the robustness and completeness of the existing thematic structure.

### Data analysis

Thematic analysis was conducted using a template analysis approach, a form of thematic analysis that emphasizes the use of hierarchical coding to organize and interpret the data [35, 36]. All interviews were audio-recorded and transcribed verbatim in simplified Chinese. To protect confidentiality, each participant was assigned a randomly generated code in place of their real name. The coding process involved four stages: (a) conduct a initial review of transcripts to identify a priori themes informed by the interview guide, alongside inductively emerging concepts; (b) develope an initial coding template; (c) systematically review and code the entire data, modify the template by inserting, removing, or merging codes as needed; and (d) finalize the comprehensive template for the entire data. The first author (XZ) and another author (SCC) independently coded the transcripts. After initial coding, overlapping and similar codes were merged to generate themes based on the objectives, and emerging themes were added to the appropriate codes. Transcripts were coded using software and manually and then merged to check for associations and patterns for comparison between participants. Coding discrepancies were resolved through discussion, with adjudication by the principal investigator (WFY) when necessary. Coding consistency was enhanced through independent coding by two researchers and iterative discussions within the research team, which supported the refinement of the coding structure and ensured analytical rigor.

### Ethics approval and consent to participate

Ethical approval for the research was granted by the Research Ethics Committee of Shandong University of Traditional Chinese Medicine Affiliated Hospital (Ethics approval number: 2023-125-KY). Participant information sheets and informed consent were obtained prior to face-to-face interviews. All study procedures were carried out in compliance with the Declaration of Helsinki and other applicable guidelines and regulations.

## Results

The interviews were conducted in Mandarin between November 2023 and May 2024. The interviews had the mean duration of 42 min. and ranged from 33 to 54 min. Our research plan encompassed a total of 12 interviews, which were subsequently augmented to 15 with the purpose of attaining thematic saturation. Table 2 presents demographics of TCM tuina practitioners. Two themes and seven subthemes were identified (Table 3).

**Table 2:** The demographics of TCM tuina practitioners

ID	Age	Gender	Work experience, years	Education level	Job title
PCT0A	30	2	3	Master	Junior
PCT0B	32	2	5	Doctor	Intermediate
M001	30	2	4	Master	Junior
M002	34	2	7	Master	Intermediate
M003	35	2	8	Master	Intermediate
M004	36	1	8	Master	Intermediate
M005	32	2	4	Master	Intermediate
M006	32	2	6	Master	Intermediate
M007	33	2	5	Doctor	Intermediate
M008	55	2	29	Doctor	Senior
M009	35	2	8	Bachelor	Intermediate
M010	49	1	15	Master	Senior
M011	36	2	12	Master	Intermediate
M012	39	2	12	Master	Senior
M013	37	2	9	Master	Intermediate

Job Title: In the Chinese medical system, job titles are classified as junior, Intermediate, and Senior, corresponding to increasing levels of clinical and academic seniority

*TCM* Traditional Chinese medicine

**Table 3:** Coding tree of the qualitative study

Themes	Sub-themes	Code Units
• Existed problems	(1) Variability in clinical practice	1) Disparities in CMT-related knowledge
		2) Variations in the selection and application of tuina manipulation 3) Variations in clinical decision-making based on experience
	(2) Barriers to parental involvement	1) Parental neglect of early CMT symptoms 2) Parental overestimation of CMT self-healing
		3) Parental insufficient awareness of long-term TCM tuina treatment needs
		4) Limited access to specialized CMT expertise
	(3) Importance of strong parental participation in TCM tuina	1) Symptom recurrence due to intermittent treatment 2) Necessity of family cooperation for relapse prevention
		3) Psychological stress during treatment (emotional burden, time, finances, distance, and family support)
		4) Challenges in performing home-assisted tuina manipulation
• Optimized approaches	(1) Promotion of public awareness	1) Popularization of knowledge about CMT (symptoms, adverse effects, and treatment)
		2) Early initiation of treatment upon CMT diagnosis
		3) Flexible application of TCM tuina in CMT treatment
	(2) Improvement of treatment protocols	1) Development of treatment protocols through parent–practitioner consultation
		2) Improvement of medical resources for CMT treatment (environment, tuina media)
		3) Emphasis on the theory of TCM
	(3) Enhancement of practitioner–parent communication	4) Provision of emotional support and accompaniment for parents
		5) Training for parents (daily care to child, posture correction, home-assisted manipulations)
		6) Adjustment of parental expectations

*Abbreviation*: *CMT* Congenital muscular torticollis, *TCM* Traditional Chinese medicine

### Theme 1: existed problems

#### Subtheme: variability in clinical practice

##### Disparities in CMT-related knowledge

Most of the TCM tuina practitioners noted that they had encountered healthcare providers who lacked adequate or accurate knowledge about CMT, which could potentially lead to misdiagnosis or suboptimal treatment strategies. One experienced TCM tuina practitioner, with over a decade of clinical practice, remarked:

*“There are few health caregivers that know a lot about CMT*,* unless it’s a specialist*,* it is hard to tell if a child has CMT.” (M012)*

##### Variations in the selection and application of TCM tuina manipulation

The interviews revealed that considerable variation among practitioners in the selection and application of tuina manipulations for treating CMT. Most practitioners in this interview emphasized that the treatment effectiveness is closely related to the appropriateness manipulation, which should be tailored to the individual characteristics and clinical presentation of each child. One practitioner highlighted that suboptimal outcomes are not necessarily due to the inappropriateness of TCM tuina therapy itself, but rather to limitations in manipulation choices, stated that :

*“The reason for ineffective results is not that it’s unsuitable for TCM tuina*,* but our manipulations may not be the best of choice*,* such as duration and frequency of manipulation. This calls for a TCM practitioner’s experience and knowledge to choose the most suitable one*,* for TCM tuina is a combination of multiple manipulations.” (M013)*

##### Variations in clinical decision-making based on experience

TCM tuina practitioners explained that their clinical decision-making was not solely based on standardized textbook knowledge, but largely influenced by their personal clinical experience. In some cases, limited diagnostic resources or insufficient emphasis on early intervention may lead to under-diagnosis or delayed treatment. As one practitioner illustrated:

*“Some children spend half a year at the basic level of TCM tuina but end up with poor results. The parents said that they have never received any facial manipulations*,* which is actually a problem of under-diagnosis because the prevention is not put forward in advance.” (M002)*

#### Subtheme: barriers to parental involvement

##### Parental neglect of early CMT symptoms

All TCM tuina practitioners emphasized that mass-type CMT involving the SCM is generally easier to diagnose, as the presence of a neck mass in newborns makes it more recognizable at birth. In contrast, non-mass type CMT is often detected only when the child begins lifting their head or sitting up. Due to its subtler symptoms, this form of CMT is frequently overlooked by parents, resulting in missed opportunities for early and timely intervention, as reported by practitioner:

*“In the process of diagnosis*,* parents often observe their child’s head mysteriously tilting*,* now to the left*,* now to the right. This is an atypical form of CMT*,* which parents might not even discern it as a disease. ”(M004)*

##### Parental overestimation of CMT self-healing

Several TCM tuina practitioners mentioned that some parents tend to believe that CMT can resolve on its own without medical intervention. However, the practitioners generally expressed skepticism toward the effectiveness of spontaneous recovery, emphasizing that delayed treatment may compromise the optimal therapeutic window and reduce the likelihood of full recovery.

*“Parents have different opinions about the after-treatment care of CMT. Some parents can be very anxious*,* while other parents think that the disease cures itself. Generally*,* parents are relatively less concerned about CMT that do not cause masses.” (M012)*

##### Parental insufficient awareness of long-term TCM tuina treatment needs

TCM tuina practitioners in the interviews observed that numerous parents, driven by anxiety for their child’s recovery, often lack a clear understanding of time commitment required for effective TCM tuina therapy in treating CMT. As a result, unrealistic expectations for rapid recovery are common. One experienced practitioner remarked:

*“There’s a gradual progress in the treatment of CMT. Some eager parents may yearn for swift healing*,* but such immediate miracles are beyond our reach. The idea that a single session of TCM tuina can dissolve massor restore muscle strength is merely a myth*,* as true healing is a process that requires patience and time. ”(M002)*

##### Limited access to specialized CMT expertise

Through communication with parents, participants found that many of them had never been exposed to any professional knowledge or guidance related to CMT prior to their child’s diagnosis. This lack of awareness often results in confusion and delays in seeking TCM tuina treatment. One tuina practitioner explained:

*“Currently*,* parents lack a specialized platform to guide them through the complexities of CMT*,* let alone to find the pathways of treatment. Their only recourse is to cast their nets into the internet*,* hoping to haul in answers amidst a sea of data.” (M003)*

#### Subtheme: importance of strong parental participation in TCM tuina

##### Symptom recurrence due to intermittent treatment

The TCM tuina practitioners stressed that consistent TCM tuina therapy is crucial for the effective treatment of CMT, as it aids in restoring normal muscle function in children. Interruptions in the treatment process often result in the recurrence of symptoms, potentially reversing the progress made and undermining the therapeutic outcomes.

*“As soon as the child’s head stops tilting with the help of the treatment*,* parents may let their guard down and stop coming to the treatment. There was a two-year-old child in the clinic with a very tilted head that had caused a cervical slip. This was because the parents thought it had been cured and treatment was no longer needed.” (M005)*

##### Necessity of family Cooperation for relapse prevention

According to the TCM tuina practitioners, even after initial recovery from CMT, children should continue to be monitored for postural abnormalities that may indicate a risk of recurrence. Ongoing parental attention and preventive care are essential in this phase to ensure lasting outcomes. One practitioner emphasized:

*“Children with CMT caused by dysplasia and malposture have a higher possibility of recurrence after treatment. In the face of this situation*,* we offer comprehensive guidance to parents for continuous after-treatment care. If their child’s head starts to tilt again*,* we strongly suggest them to come back for a review.” (M006)*

##### Psychological stress during CMT treatment (emotional burden, time, finances, distance, and family support)

Most of the TCM tuina practitioners observed that the parents often face significant psychological stress during their child’s treatment for CMT, particularly during passive stretching procedures. As the child is placed in a passive position and may respond with intense crying, parents must simultaneously manage their emotional reactions and uphold their caregiving responsibilities. One practitioner recounted:

*“During the treatment process*,* emotional parents could not help crying looking at their children receive the treatment. They know it well that they have to go through the difficult time to get their children cured” (M009)*

Another practitioner further explained:

*“Some children cry a lot when given TCM tuina. Out of parental love*,* parents may discontinue the process because it’s too hard for them to watch their kids cry. Some of our tuina manipulations are not that gentle*,* which may also cause grandparents to intervene the process. It’s just too cruel for them to watch their grandkids cry like this. So the results of treatment can also be affected by this.” (PCT0B)*

##### Challenges in performing home-assisted tuina manipulation

Although opinions vary on whether parents should perform home-assisted manipulations at home, the common view among TCM tuina practitioners is that these home-assisted manipulations are not easy for parents to carry out when they are done independently at the beginning.

*“Parents have no idea how much pressure is applied in the process for the technique can not be quantified*,* which concerns them the most. For diseases that have caused masses*,* continuous after-treatment care is needed back home. However*,* parents should pay attention to the pressure they apply in the process*,* because too much pressure will cause muscle hematoma*,* which may get worse the next day*,* finally leading to swelling muscles. That is a therapeutic counterproductive problem we hate to see.” (M005)*

### Theme: optimized approaches

#### Subtheme: promotion of public awareness

##### Popularization of knowledge about CMT symptoms

Educating the public about the symptoms of CMT is crucial in promoting early detection and intervention. By recognizing CMT signs, individuals and their families can seek timely medical evaluation and appropriate treatment.

*“When the health caregivers detected the existence of the disease*,* they will first take the initiative check at the infants at the maternity hotels*,* which makes way for early examination. Some parents found that their children’s one side of eyes or faces is bigger than the other side*,* or that their head starts tilting*,* which makes them come to us for advice.” (M013)*

##### Early initiation of treatment upon CMT diagnosis

As described by the TCM tuina practitioner, the primary affected structures in CMT are the neck muscles, particularly the SCM, which are closely related to children’s growth and development. Given that muscle growth and adaptability are strongly influenced by early-life development, timely initiation of treatment is considered essential for enhancing the likelihood of restoring affected muscles to normal function and structure while minimizing long-term complications.

*“As for the timing of the intervention*,* I must emphasize that the sooner*,* the better. For instance*,* should the signs of CMT be identified within the first month of a newborn’s life*,* we leap into action without delay*,* setting the stage for the swift dissolution of the SCM mass. I recall a child under my treatment who exhibited a remarkable pace of recovery*,* the mass had vanished*,* fully absorbed*,* within the fleeting span of just one month. ”(M011)*

##### Flexible application of TCM tuina in CMT treatment

In certain instances, TCM tuina may serve as an independent therapeutic modality. However, it is often integrated into a holistic treatment strategy, where it complements additional interventions including surgery and therapeutic exercise, thereby contributing to a multifaceted approach to patient care.

*“Some children are not totally cured after the surgery*,* they might still have muscle contracture. Under these circumstances*,* it is recommended that they come back to us for TCM tuina for a week or a month to take care of the surgery wound. ”(M002)*

#### Subtheme: improvement of treatment protocols

##### Development of treatment protocols through parent–practitioner consultation

In the TCM tuina practitioners’ opinion, the participation of parents in the formulation of the treatment protocol has a positive influence on the implementation of TCM tuina. It can improve patient’s adherence. Also, parents can give timely feedback on the symptoms and treatment effects of their children, which makes the treatment procedures more personalized.

*“If the masses caused different sizes of SCM*,* we strongly suggest them to come in for consolidation therapy once a week or twice a week. The treatment can be terminated if the parents think it is cured enough. It is up for parents to decide to take the treatment or not.” (M013)*

##### Improvement of medical resources for CMT treatment (environment, tuina media)

TCM tuina practitioners recognized the importance of a comfortable and segregated therapeutic room to enhance treatment efficacy, reduce anxiety and cross-infections risks. Additionally, enriching the tuina medium with herbal extracts, essential oils, or analgesics can provide targeted relief for specific symptoms associated with CMT.

*“I certainly think it would be ideal if there was a segregated therapeutic room for children with CMT*,* because there is a possibility for cross-infections between them and other children. Children with CMT may come for TCM tuina for a few more days and then catch a cold or have a fever after treatment.” (M012)*

##### Emphasis on the theory of TCM

The TCM tuina practitioners emphasized the importance of integrating TCM theory into the understanding and treatment of CMT. They advocated for formulating personalized treatment plans based on TCM principles, such as stimulating specific acupuncture points and meridians to regulate the flow of “qi” and support the recovery process.

*“CMT is not just a problem of the neck muscles*,* but a problem of general health*,* which may be caused by anemia or the lack of “qi”. Generally*,* we use TCM tuina of internal medicine to improve the muscle strength*,* which actually alleviates the problem of CMT. The key is to strengthen the spleen and nourish the blood.”(M012)*

#### Subtheme: enhancement of TCM tuina practitioner-patient communication

##### Provision of emotional support and accompaniment for parents

TCM tuina practitioners believed that the companionship and support given to parents during treatment as crucial for maintaining parental commitment to therapy. By establishing a close, empathetic relationship and offering consistent emotional support, they strengthen treatment adherence, which is essential for improving outcomes.

*“Parents tend to be very anxious when they found out about CMT*,* which is partly caused by the information on the internet. They come to us with tons of questions*,* like “is it curable?”*,* or “how long does it take?”. We have to take care of the parents’ feelings while applying treatment on the children.” (M008)*

##### Training for parents (daily care to child, posture correction, home-assisted manipulations)

The TCM tuina practitioners emphasized that effective CMT treatment extends beyond clinical sessions and requires active parental involvement in daily care and home-based interventions. To this end, they routinely provide parents with training on essential aspects such as posture correction and home-assisted tuina manipulations, always prioritizing safety.

*“We teach the parents about the* tuina manipulations, *because after-care treatment is needed back home for CMT with mass*,* which can be done after the children fell asleep. Three times a day should be used for bigger mass for once eight hours or twice/three times a day. This works better cooperated with our work.” (M013)*

##### Adjustment of parental expectations

During the course of TCM tuina treatment for CMT, a temporary plateau in symptom improvement is not uncommon. This bottleneck can lead to heightened parental anxiety, which is often recognized by experienced practitioners. In response, timely and effective communication is essential to help parents recalibrate their expectations and maintain confidence in the therapeutic process.

*“We try to keep the parents in the loop about the treatment. The parents are free to check the process of the treatment. After a week or a month*,* we are able to tell the results of the masses.” (M009)*

## Discussion

### Main findings

This qualitative study explored the clinical application of TCM tuina in managing CMT in pediatrics from the perspectives of frontline practitioners in China. Two primary themes emerged: current challenges and optimization strategies. First, numerous challenges exist in clinical practice, including variability in treatment selection and manipulation techniques, disparities in practitioner knowledge, and inconsistencies in clinical decision-making. Practitioners reported widespread parental barriers, such as neglect of early CMT symptoms, overestimation of spontaneous resolution, limited understanding of long-term treatment requirements, and significant psychological and logistical burdens. The necessity of active family cooperation was emphasized to ensure treatment continuity and prevent recurrence. Second, practitioners proposed optimized strategies to improve outcomes, including enhancing public awareness of CMT and its treatment, initiating therapy promptly after diagnosis, and flexibly applying TCM tuina tailored to individual needs. They advocated for standardized treatment protocols developed collaboratively with parents, improvements in clinical resources and environments, and adherence to traditional TCM theoretical principles. Furthermore, emotional support for parents, hands-on training in home care, and managing expectations through effective communication were considered crucial for successful therapy.

### Differences and novel contributions

Unlike prior researches that primarily assessed therapeutic outcomes, our study emphasizes the perspectives of frontline TCM tuina practitioners, lighting on their clinical reasoning, practical challenges, and treatment approaches in managing CMT. (1) Although guidelines for CMT are available, our findings suggest that diagnostic practices among TCM tuina practitioners remain subjective and variable, revealing key factors that affect the practical application of tuina in pediatric settings.TCM tuina practitioners often diagnosed CMT without fully adhering to established diagnostic criteria, typically focusing only on SCM masses, craniofacial asymmetry, and neck range of motion, despite guidelines recommending seven structural measurements for comprehensive assessment [37]. They often rely on visual and experiential assessments, a method akin to that previously observed among physiotherapists [38]. However, the available studies did not find a correlation between visual assessment of CMT and clinical experience, and this type of assessment lacks accuracy [39]. (2) Our findings underscore that effective communication and collaboration among practitioners and caregivers play a critical role in facilitating the practical application of TCM tuina—a dimension that remains underexplored in current CMT literature. First, effective practitioner–caregiver communication is essential. At the initial consultation, practitioner should offer a systematic explanation of the pathophysiology, clinical classification, expected course of treatment, and recommended home care practices for CMT to establish caregivers’ foundational understanding of the condition. For home-based interventions that rely on parental execution—such as postural training and manual techniques—real-time demonstrations during clinic visits, supplemented by standardized instructional videos, can improve technical accuracy and enhance parents’ perceived safety and confidence [40–42]. When caregivers express concerns regarding the safety or efficacy of tuina therapy, practitioners should adopt active listening strategies and provide empathic, evidence-based responses to address emotional distress and reinforce trust. Second, communication within the family unit also plays a critical role. Study has shown that extended family members—such as grandparents—often play a caregiving role, and their attitudes can either reinforce or hinder adherence [43]. To address this, practitioners may encourage families to clarify caregiving roles, for instance, through family meetings to define responsibilities, and when necessary, involve all primary caregivers in treatment planning. Sustained familial support and adaptive communication strategies can ultimately foster a more cohesive and responsive caregiving environment, thereby promoting adherence and improving therapeutic outcomes [44]. (3) This study moves beyond previous general suggestions on integrating TCM tuina with physical therapy by offering specific, feasible strategies such as joint assessments and culturally adapted care planning. Many low-risk techniques in TCM tuina, such as pushing and kneading, align with manual therapies already practiced by physical therapists and can be safely performed by trained healthcare providers [15, 45]. In contrast, high-risk manipulations like pulling, stretching should be reserved for clinicians with specialized anatomical knowledge and CMT-specific training [46]. For practitioners unfamiliar with TCM philosophy, the holistic goal of improving muscle strength may be translated into culturally appropriate interventions, such as enhancing pediatric nutrition [47]. Additionally, integrating tuina with widely accepted non-invasive physical therapy modalities (e.g., therapeutic exercise, hot compresses) may provide synergistic benefits [14, 15]. Establishing collaborative treatment pathways involving both TCM tuina practitioners and physical therapists—for joint assessment, goal setting, and treatment delivery—could help optimize individualized care for children with CMT in diverse healthcare settings. (4) Our study highlights a key factor affecting the application of TCM tuina for CMT in children: limited parental awareness of early symptoms such as facial asymmetry and restricted neck movement. Despite existing evidence emphasizing the importance of early detection [48–51], practitioners rarely adopt proactive strategies to educate caregivers. This lack of engagement delays timely intervention and undermines the effectiveness of tuina. Strengthening parental education and early screening could therefore play a critical role in improving clinical outcomes and expanding the effective use of TCM tuina in pediatric CMT management.

### Implications for clinical practice

The findings of this study have several implications for clinical practice: (1) Treatment plans and training: More than half of maternal and child health hospitals in China have TCM departments, which play a vital role in the nation’s healthcare system [28]. Therefore, it is critical for the healthcare workers in the TCM hospital to promote continuous training to improve the standard of pediatric care [52].Our study found that treatment plans were often shaped by TCM tuina practitioners’ personal preferences and limited familiarity with the full range of available CMT modalities. This is concerning given the existence of regularly updated clinical practice guidelines for CMT—revised every five years—which are critical for promoting early detection and timely intervention in pediatric care practice [53]. Greater efforts are needed to disseminate these guidelines among tuina practitioners to ensure evidence-informed and standardized care for children with CMT. (2) Promoting adherence: TCM tuina practitioners highlighted that parental adherence is crucial to the effective application of tuina for CMT, as it directly influences treatment outcomes,, consistent with findings from previous studies [54]. For CMT, adherence includes attending outpatient visits punctually and performing home posture exercises. Timely attendance at appointments is particularly important in managing CMT, as consistent treatment sessions significantly enhance intervention efficacy, thereby increasing the likelihood of a cure and reducing the risk of recurrence [55]. (3) Multidisciplinary collaboration: Integrating TCM tuina with conventional physical therapy techniques (e.g., therapeutic exercise, hot compresses) could yield synergistic benefits. Studies have shown that narrow focus contributed to a restricted range of treatment options and a lack of therapeutic diversity [56, 57]. Consequently, we emphasize the rational application of TCM tuina based on the pediatric actual condition, and advocate for the integration of multiple approaches to ensure more comprehensive and individualized treatment [37, 58, 59].

### Guidance for future research

Several areas warrant further investigation: (1) Objective diagnostic tools: Future research should explore the integration of objective tools, such as ultrasonography and shear wave elastography, to enhance diagnostic accuracy for CMT, particularly in mass-type cases. In instances of CMT where a mass is not present on the SCM, the diagnosis tends to be significantly delayed or even overlooked, in contrast to cases of CMT with a palpable mass [60]. Such no-mass type cases often remain undiagnosed until the child reaches at least five years of age [61] —a trend consistent with the findings of our study. Studies have shown that ultrasonography can assist in the initial diagnosis and severity assessment of CMT in infants, irrespective of the presence of an SCM mass [62, 63]. Fibrosis of the SCM associated with CMT can also be quantitatively and objectively assessed by measuring shear wave velocity [64, 65]. (2) Standardization of home exercises: Well-designed studies are needed to establish standardized, evidence-based home exercise protocols, specifying optimal content, frequency, and duration for effective CMT management. Our study found that it is important for incorporating home exercises into the CMT treatment regimen to enhance outcomes. However, there is currently no consensus on the specific program or frequency for these home exercises [37, 66].

### Strengths

To our knowledge, there is limited qualitative research specifically explored factors influencing the application of TCM tuina for CMT from the perspective of TCM tuina practitioners working in hospital settings. The practitioners in this study came from two regions, Jinan (Shandong, China) and Shenzhen (Guangdong, China), which allowed a more diverse information to be collected. More than half of participants were from grade A tertiary hospitals with specialist CMT clinics, which have rich experience in diagnosis and treatment of CMT classification and symptoms. This was advantageous in shaping the results of the study. All interviews were conducted in a face-to-face, individual format, which provide a conducive environment for participants to freely express their views and delve into topics in-depth, without the presence of group pressure or external influences that could potentially impact their responses.

### Limitations

First, all participants came from grade A tertiary hospitals in China, which might not have represented the experience from hospitals of other grades, especially in rural and remote areas where there is no better medical level. Second, some views on parents were collected from tuina practitioners, but we did not validate such findings from parents. Nevertheless, TCM tuina practitioners’ experience in communicating with parents still gives insight into the common problems and barriers encountered by the parents. In addition, since interviewers only recruited those with whom they had a good relationship, which could have led to a lack of credibility. Finally, the main authors have a clinical background in TCM, which may have unintentionally shape the interview interactions in a more academic or theory- driven direction. Additionally, since the researchers was involved in both data collection and analysis, there is a risk of confirmation bias.

## Conclusion

Based on the findings of this qualitative study, the clinical application of TCM tuina for children with CMT is influenced by a range of practitioner and parent-related factors. Existing challenges—such as inconsistencies in clinical practice, limited parental awareness, and inadequate family engagement—hinder treatment effectiveness and continuity. However, experienced tuina practitioners also proposed practical and culturally grounded strategies to optimize care, including early intervention, individualized techniques, standardized protocols, and strengthened parent-practitioner collaboration. These insights underscore the need for integrated efforts to enhance practitioner training, public education, and family support, ultimately promoting the broader adoption and effectiveness of TCM tuina in pediatric CMT management.

## Supplementary Information

Supplementary Material 1.

Supplementary Material 2.

## Data Availability

The original contributions presented in the study are included in the article/supplementary material. Further inquiries can be directed to the corresponding authors.

## Abbreviations

CMT: Congenital muscular torticollis
SCM: Sternocleidomastoid muscle
TCM: Traditional Chinese medicine
COREQ: Consolidated Criteria for Reporting Qualitative Research
