# Supplementary material for: Understanding factors related to application of traditional Chinese medicine tuina for congenital muscular torticollis in children: a qualitative study based on traditional Chinese medicine tuina practitioners
Source: BMC Complement Med Ther. 2026 Jan 10;26:49. doi: 10.1186/s12906-026-05246-0 (PMC12882261; doi:10.1186/s12906-026-05246-0)
Supplement: Supplementary file 1 — Supplementary Material 1. [file 12906_2026_5246_MOESM1_ESM.pdf]

伦理审查批件

<table>
<tr><td>批件号</td><td colspan="3">(2023)伦审第(125)号-KY</td></tr>
<tr><td>项目名称</td><td colspan="3">医生运用小儿推拿疗法改善小儿肌性斜颈的效果与经验</td></tr>
<tr><td>项目来源</td><td colspan="3">自拟课题</td></tr>
<tr><td>研究单位</td><td colspan="3">山东中医药大学附属医院</td></tr>
<tr><td>主要研究者</td><td colspan="3">丁菲菲</td></tr>
<tr><td>审查类别</td><td>初始审查</td><td>审查方式</td><td>快速审查</td></tr>
<tr><td>审查日期</td><td>2023-11-20</td><td>审查地点</td><td>伦理委员会会议室</td></tr>
<tr><td>审查委员</td><td colspan="3">曹永福,陈柏楠</td></tr>
<tr><td>批准文件</td><td colspan="3">1.初始审查申请
2.研究者经济利益声明
3.临床研究方案(版本号 v1.0_版本日期 2023-08-01)
4.知情同意书(版本号 v1.0_版本日期 2023-08-01)
5.招募受试者的材料(版本号 v1.0_版本日期 2023-08-01)
6.提供给受试者的书面文件(版本号 v1.0_版本日期 2023-08-01)，如日记卡、调查问卷等
7.病例报告表
8.研究者手册
9.主要研究者专业履历、GCP 证书
10.研究人员名单、执业资格及其研究职责分工
11.临床研究中心立项批件</td></tr>
</table>

审查意见

根据国卫科教《涉及人的生命科学和医学研究伦理审查办法（2023）》、《医疗器械临床试验质量管理规范（2022）》、国家药监局《药物临床试验质量管理规范（2020）》、WMA《赫尔辛基宣言》和 CIOMS《人体生物医学研究国际道德指南》等伦理原则，经本伦理委员会审查，同意按批准文件开展临床试验/研究。

研究开始前，请研究者完成临床试验注册及医学研究登记备案。

请遵循 GCP 原则、遵循伦理委员会批准的方案开展临床研究，保护研究参与者的健康与权利。

凡涉及中国人类遗传资源、需报批的研究项目，应获得中国人类遗传资源管理办公室批准后才能开始研究，且遗传办批件应提交本中心伦理委员会备案。对于跨国/境研-究的开展，应充分考虑并遵循研究所在国/境的法律、法规、政策和指南，以及当地的社会文化特点，做好受试者保护工作。

研究者将接受伦理委员会的现场核查与监督，研究过程中的审查：

如变更主要研究者，对临床研究方案、知情同意书、招募材料等，应及时提交修正案审查申请，重新审查并获得批准后执行。

发生严重不良事件，请申请人及时提交严重不良事件报告。

请按照伦理委员会规定的年度/定期跟踪查频率，申请人在截止日期前 1 个月提交研究进展报告，若超出有效期未递交《研究进展报告》及获得伦理审查同意，研究者必须立即停

止所有研究活动，包括干预措施和数据收集。申办者应当向组长单位伦理委员会提交各中心研究进展的汇总报告；当出现任何可能显著影响试验进行或增加受试者危险的情况时，请申请人及时向伦理委员会提交书面报告。

研究纳入不符合纳入标准或符合排除标准的受试者，符合中止试验规定而未让受试者退出研究，给予错误治疗或剂量，给予方案禁止的合并用药等没有遵从方案开展研究的情况；或可能对受试者的权益/健康以及研究的科学性造成不良影响等违背 GCP 原则的情况，请申办者/监查员/研究者提交违背方案报告。

申请人暂停或提前终止临床研究，请及时提交暂停/终止研究报告。

研究结束后，研究者提交结题报告。

年度/定期跟踪审查频率	12 个月
有效期	2023-11-20 至 2024-11-20
联系人与联系电话	袁杰 0531-68616733
主席签字	
伦理委员会	山东中医药大学附属医院伦理委员会 （盖章）
日期	2023-11-20
